# Tinnitus disorder and musical auditory training: a double-blind randomized controlled clinical trial

**DOI:** 10.1590/2317-1782/e20250150en

**Published:** 2026-06-15

**Authors:** Christine Grellmann Schumacher, Katya Guglielmi Marcondes Freire, Denis Altieri de Oliveira Moraes, Michele Vargas Garcia, Dayane Domeneghini Didoné

**Affiliations:** 1 Departamento de Fonoaudiologia, Centro de Ciências da Saúde, Universidade Federal de Santa Maria – UFSM - Santa Maria (RS), Brasil.

**Keywords:** Tinnitus, Auditory Perception, Hearing, Controlled Clinical Trial, Evoked Potentials, Auditory

## Abstract

**Purpose:**

To evaluate the effect of Musical Auditory Training (MAT) on auditory system neuroplasticity and on tinnitus perception in adults.

**Methods:**

A randomized double-blind clinical trial, approved by the Research Ethics Committee. Twenty adults with tinnitus complaints for at least six months, intensity greater than four points on the Visual Analog Scale (VAS), normal bilateral hearing thresholds, and preserved neural synchrony at the brainstem level were included. Participants were randomly assigned to two groups: Control Group (CG, n=10), submitted to passive stimulation with audiovisual material without tasks, and Study Group (SG, n=10), submitted to MAT. Both groups underwent eight sessions, twice a week, over a four-week period. Assessments included verbal Long-Latency Auditory Evoked Potentials (LLAEP), to investigate neuroplasticity, as well as the VAS (volume and discomfort) and the Tinnitus Handicap Inventory (THI), to measure symptom perception.

**Results:**

Both groups showed improvement in tinnitus perception after the intervention. However, a differentiated analysis revealed that the CG presented restricted changes, with improvement only in the duration of the P3 component of the LLAEP. In contrast, the SG showed broader changes, including significant alterations in both amplitude and duration of P3, suggesting greater neural recruitment associated with auditory processing. In addition, only the SG demonstrated consistent reductions in tinnitus volume and discomfort, as well as in the impact on quality of life, as reflected in THI scores.

**Conclusion:**

After four weeks, MAT proved to be superior to passive stimulation, promoting positive changes in auditory neuroplasticity, a greater reduction in tinnitus intensity and annoyance, and a significant improvement in participants’ quality of life.

## INTRODUCTION

Tinnitus disorder is a multifaceted symptom arising from multiple mechanisms. In 2021, the scientific literature proposed a conceptual differentiation between the terms “tinnitus” and “tinnitus disorder,” whereby tinnitus is limited to the isolated auditory perception, and tinnitus disorder includes emotional, cognitive, and behavioral aspects, frequently associated with psychological distress, functional disorders, and behavioral changes^(1)^.

The literature indicates that tinnitus disorder is not restricted to peripheral dysfunctions of the auditory system but involves central alterations that reflect changes in the neuroplasticity of the auditory system, generating disorganization in the central auditory pathways. These alterations significantly impact the organization of the auditory pathways, modifying neural processing^(2)^.

Among the most accepted etiological hypotheses, the role of central neuroplastic changes stands out. Individuals with tinnitus present increased activity in the auditory pathways and impairment in cortical areas related to cognitive and emotional functions, such as the limbic and cognitive systems^(3)^. In this context, strategies such as auditory training have been investigated, considering their capacity to promote neural reorganization through acoustic stimulation and cognitive and metacognitive strategies^(4)^.

Thus, musical auditory training emerges as a potentially effective approach. This training stimulates not only auditory abilities but also cognitive functions, such as attention and working memory^(5,6)^.

Among the tools used to evaluate the effects of auditory intervention, Long-Latency Auditory Evoked Potentials (LLAEP) stand out for providing detailed information about the activity of the Central Auditory Nervous System, including auditory discrimination and auditory memory abilities^(7)^. Alterations in these potentials, frequently observed in individuals with tinnitus disorder, reflect reorganizations in subcortical, temporoparietal, prefrontal, and limbic neural networks, compromising both auditory and cognitive functions^(8)^.

Although auditory training has been identified as a promising strategy to enhance neural plasticity in individuals with tinnitus disorder, the literature lacks investigations exploring its effectiveness. In particular, the application of musical auditory training in this context has not yet been widely studied.

Given this gap, this study is based on the hypothesis that musical auditory training may promote the reorganization of central auditory pathways in individuals with tinnitus disorder, resulting in favorable neural alterations and improvement in symptom perception. Thus, the objective of this study was to evaluate the effects of MAT in adults with tinnitus disorder, considering both the functional aspects of central auditory processing and the clinical repercussions of the symptom. The innovative character of this investigation lies in the combined use of MAT and LLAEP, allowing an integrated understanding of both the subjective aspects of symptom perception and the objective correlates of neural activity. By simultaneously exploring these dimensions, this study contributes innovative evidence to the clinical management of tinnitus, offering a non-invasive, low-cost intervention with broad potential application in speech-language and hearing practice.

## METHOD

Randomized double-blind controlled clinical trial, registered on the Clinical Trials platform (NCT06371287), based on the CONSORT checklist, approved by the Research Ethics Committee under number 64696022.1.0000.5346. All procedures were carried out at the audiology and auditory electrophysiology outpatient clinic of the Speech-Language Pathology Service of the Federal University of Santa Maria. All individuals involved signed the Informed Consent Form.

Participant recruitment took place between April 2023 and February 2024. The intervention period lasted four weeks, with reassessment conducted four weeks after the end of the sessions.

The study comprised two groups: one that received intervention through Musical Auditory Training and another, the control group, with passive intervention. The distribution of subjects occurred randomly, in which they were randomized between the two groups. Randomization was performed by an independent researcher who allocated the subjects in an Excel spreadsheet in alphabetical order and conducted the draw of the intervention according to the nominal order; that is, the draw was performed for each subject allocated in the spreadsheet. Allocation concealment was ensured, since the independent researcher only disclosed group assignments to those responsible for the intervention after the completion of the initial assessments. The study was characterized as double-blind, since participants were not aware of the type of intervention received. In addition, the researchers responsible for the initial and final evaluations, as well as the judges who analyzed the electrophysiological recordings, remained blinded regarding group allocation. Only the researcher responsible for applying the interventions had access to the designation, and did not participate in the evaluation stages or in the data analysis.

Considering the sample size calculation to verify the difference in the mean score between the two assessments, before and after, in paired data per subject, the following parameters were established: effect size equal to 0.8 deviations, nominal significance level (probability of type I error) equal to 5%, and test power of 75%. Thus, the minimum number of individuals in each group should be at least 10 individuals.

The following inclusion criteria were established:

- Individuals of both sexes aged between 18 years and 55 years;
- Complaint of tinnitus disorder;
- Discomfort score of at least four on the Visual Analog Scale, considered a moderate level of symptom discomfort, characterizing tinnitus disorder;
- Minimum perception of six months of tinnitus disorder in both ears^(9)^;
- Previous consultation with an otorhinolaryngologist;
- Hearing thresholds within normal limits bilaterally or sensorineural hearing loss up to mild degree in the quadritonal average (500, 1000, 2000, and 4000 Hz)^(10)^;
- Type A tympanometric curve bilaterally;
- Normality in the Auditory Brainstem Response (ABR)^(11)^, indicating integrity of the auditory system;
- Normal performance in the Mini-Mental State Examination^(12)^.

The exclusion criteria were:

- Speech alterations, diagnosed or apparent psychiatric or neurological diseases;
- History of cranial or brain trauma;
- Presence of somatosensory and vascular tinnitus modulation, detected through the Screening for tinnitus of vascular origin^(13)^ and the Somatosensory modulation test of tinnitus^(14)^;
- Presence of symptoms and/or diagnosis of external or middle ear impairment^(15)^;
- Having initiated a new treatment (pharmacological or therapeutic) or having been diagnosed with any disease of any origin in the last month;
- Undergoing another intervention for tinnitus during the study;
- Score higher than 11 points on the Hospital Anxiety and Depression Scale (HADS), indicating moderate risk of anxiety or depression^(16)^.

The procedures selected for this study were defined according to the sample composition. These were performed at two different moments by the same examiner. Initially, the subject attended to undergo tinnitus assessment procedures, questionnaires, and basic audiological evaluation (one hour duration), and at another moment for electrophysiological evaluation (one hour duration). The order of assessment procedures was as follows: anamnesis, visual inspection of the external acoustic meatus, pure-tone audiometry, speech audiometry, immittance testing, and application of the questionnaires Tinnitus Handicap Inventory (THI) and Visual Analog Scale (VAS). For electrophysiological evaluation, the Auditory Brainstem Response (ABR) was initially performed to verify the integrity of the auditory pathway, followed by the Long-Latency Auditory Evoked Potentials (LLAEP). Subsequently, the selected subjects were submitted to the intervention (MAT or passive stimulation) and later reassessed through LLAEP to analyze the outcome of the applied interventions.

### Long-Latency Auditory Evoked Potentials (LLAEP)

Long-Latency Auditory Evoked Potentials (LLAEP) were used to evaluate neuroplasticity before and after four weeks of intervention. Measurements were performed with the SmartEP (Intelligent Hearing Systems), using insert earphones and following protocols to minimize artifacts. Preparation included skin cleaning with abrasive paste (NUPREP) and fixation of the electrodes (active electrode at Cz, ground at Fpz, and reference electrodes positioned on the left (A1) and right (A2) earlobes) with electrolytic paste and adhesive tape. Recordings were obtained using an oddball paradigm (/ba/ and /di/), with alternating polarity, intensity of 80 dBnHL, and 300 stimuli (240 BA and 60 DI). Strict criteria were followed for artifact control and parameters, such as rate of 1.10/s, recording window of 510 ms, gain of 100K, filter of 100–3000 Hz, electroencephalogram (EEG) window of 31%, and impedance of a maximum of 3 KΩ. Artifacts were monitored to not exceed 10% of the number of stimulations, maintaining a signal-to-noise ratio ≥ 1 dB and residual noise ≤ 0.11 dB.

Two blinded expert judges analyzed the latency of waves P1, N1, P2, N2, and P300^(17)^. There was no disagreement in the markings. It is noteworthy that amplitude was considered according to the morphology of the components, without considering minimum reference parameters, since the objective of the study was to measure neuroplastic activity. Standardization ensured the reliability of the results. After marking the LLAEP components by the expert judges, the duration of the P3 component was calculated considering the beginning and the end of the wave. The beginning of the wave was considered at the initial point of the valley and the end at the termination of the valley of the wave. Duration was calculated by subtracting the value in milliseconds of the end and the beginning of the P3 wave. The calculation was performed by applying a subtraction formula in Excel spreadsheets.

### Tinnitus Handicap Inventory (THI)

The Tinnitus Handicap Inventory (THI) was used to measure the influence of tinnitus disorder on the quality of life of the participants and to classify them into severity levels based on the total score^(18)^. Composed of 25 questions, the questionnaire evaluates tinnitus perception and its functional, emotional, and catastrophic impact. Participants answered the questions by choosing between “yes,” “no,” or “sometimes,” according to the relevance to their experience. The obtained score allowed classification into five degrees: slight, mild, moderate, severe, and catastrophic. The THI was applied both in the initial assessment and in the reassessment, conducted after four weeks of intervention, to analyze changes in perceived impact.

### Visual Analog Scale (VAS)

The Visual Analog Scale (VAS) is widely used to measure chronic pain and, in individuals with tinnitus disorder, evaluates the degree of discomfort caused by the symptom^(19)^. It is a subjective measure in which participants assign a score from 0 to 10, considering both the perceived discomfort (VAS D) and volume (VAS V) of tinnitus. For characterization of the disorder, only subjects with VAS scores greater than or equal to 4 were included^(19)^. In addition, the VAS was applied before and after the intervention, carried out over four weeks, to monitor changes in symptom perception.

### Intervention procedures

#### Study Group – Musical Auditory Training

The musical training was based on the protocol proposed by Freire et al.^(6)^, originally developed for older adults with hearing aids and adapted in this study as an intervention for tinnitus. The protocol aims at auditory training through the hierarchical organization of auditory abilities associated with musicality.

Eight sessions were conducted, distributed over four weeks, with two weekly sessions lasting 40 to 50 minutes. Subjects used supra-aural headphones calibrated to the individual auditory comfort level at the beginning of each session. The activities trained abilities such as figure-ground for instrumental sounds, directed listening, duration, frequency, rhythm, auditory closure, and audiovisual memory, with emphasis on temporal processing, working memory, and selective attention, organized in increasing levels of difficulty.

Each session included 10 exercises per level, adjusting complexity according to the time and nature of the activities. Monaural activities began with the left ear, considering the predominance of the right hemisphere in processing non-verbal stimuli. The protocol, available online, was self-explanatory, with in-person monitoring by the researchers, and included clear instructions regarding the objective of each task, adapting response time to promote working memory stimulation.

It is noteworthy that the researchers had access to the complete version of MAT and to the data on the percentage of correct responses for each session, available on the website. Access was authorized and granted by the responsible author.

#### Control Group – Passive intervention

To minimize bias and evaluate the effectiveness of Musical Auditory Training (MAT) on the neuroplasticity of the auditory system and the perception of tinnitus disorder, a group with passive intervention was included. This group performed eight sessions over four weeks, with two weekly sessions, under conditions identical to those of the study group (40 to 50 minutes per session), accompanied by the researchers.

The passive intervention used instrumental music (Sonata for Two Pianos in D Major, K448, by Mozart) together with the presentation of silent films, providing visual and auditory stimulation. The selected films included works such as *Cirque Du Soleil: Journey of Man* and films by Chaplin (*Modern Times*, *The Great Dictator*, *The Gold Rush*, and *Limelight*), presented in random order^(6)^.

This approach aimed to evaluate the impact of musical exposure without the structuring of auditory abilities, in comparison with MAT. The volume of the music was individually adjusted so as not to mask the tinnitus.

## SAMPLE

The sample consisted of 20 subjects with tinnitus disorder, 10 in the Control Group and 10 in the Study Group. In this study, 70 subjects contacted the researchers to participate in the research, and only 20 met the study criteria. The other 50 subjects were excluded based on specific criteria, including ten due to scores higher than 11 on the HADS, eight due to age, nine due to hearing loss greater than mild degree, seven due to psychiatric or neurological diseases, seven due to middle ear impairment, four due to VAS scores lower than four points, and five due to the presence of modulation in screening tests of the somatosensory and/or vascular component. It is noteworthy that there were no missing data or segment losses during the conduction of the longitudinal research.

**Figure 1:**
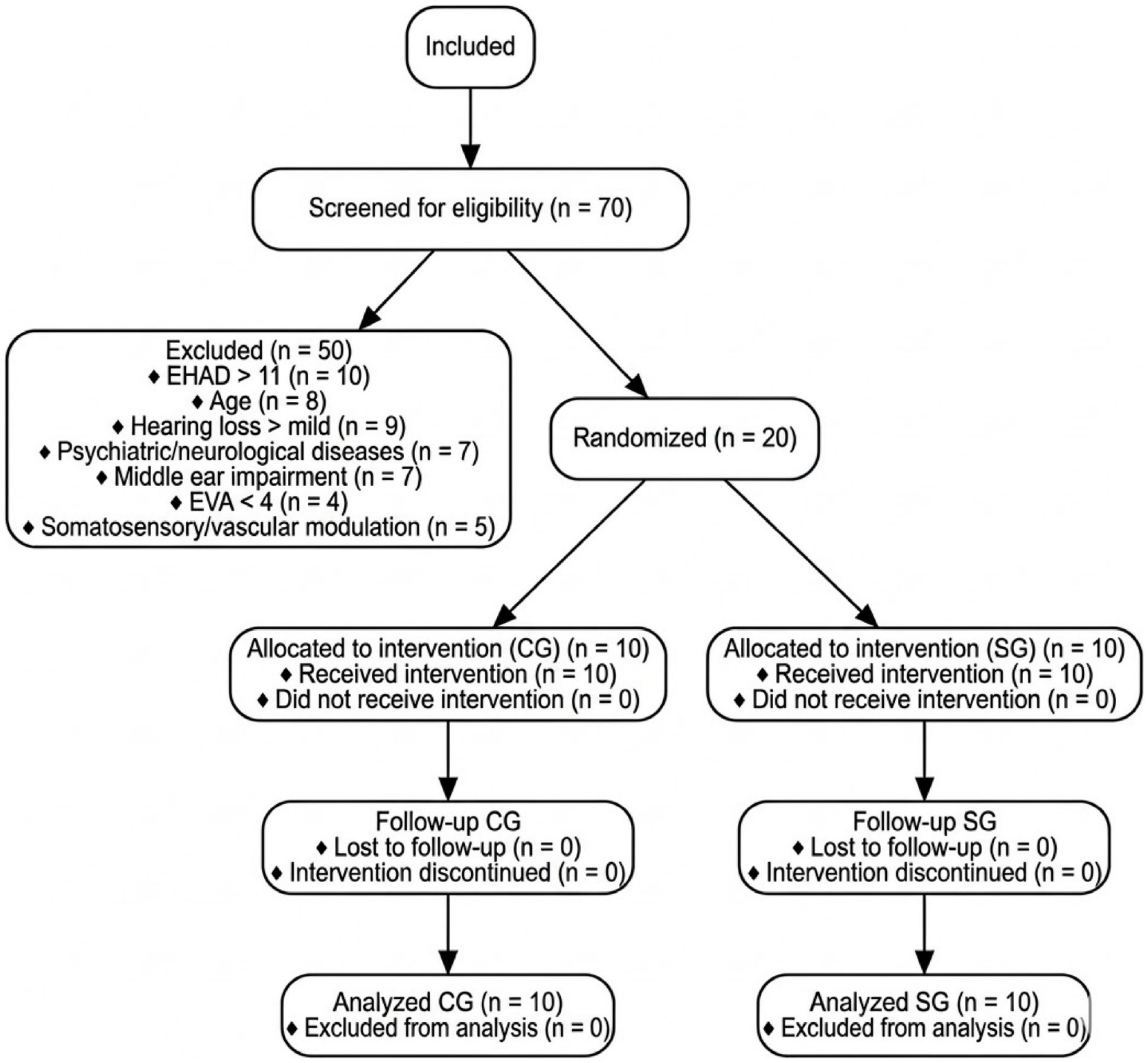
Flowchart

The sample flowchart of the study can be visualized in Figure 1.

**Caption:** n = total number of subjects; HADS = Hospital Anxiety and Depression Scale; VAS = Visual Analog Scale; CG = Control Group; SG = Study Group

The primary outcome of the study was tinnitus perception and the reorganization of central auditory pathways after the application of MAT. Secondary outcomes included the measurement of tinnitus volume and discomfort, as well as the latency, amplitude, and duration of LLAEP after the applied interventions.

The results were tabulated in Microsoft Office Excel for analysis. A significance level of 5% was used, and analyses were performed using the R software.

Data were tested for normality using the Shapiro–Wilk test, while the Levene test was used to evaluate homogeneity of variances between groups. VAS, THI, and LLAEP latency, amplitude, and duration variables were compared between right ear and left ear, and between groups using the independent samples t-test, at pre- and post-intervention moments.

When groups were analyzed pre- and post-intervention, statistical analysis was performed using the paired samples t-test. For subsequent analyses, the mean between the right ear and the left ear was considered, since there was no statistically significant difference between the evaluated sides in both groups.

## RESULTS

Six female participants and four male participants were included in the CG (n=10). In the SG, five female and five male subjects were included (n=10). The comparison between the groups did not show a statistically significant difference (p-value=0.653).

Data regarding age, education level, duration of perception, pre-intervention VAS V, pre-intervention VAS D, and pre-intervention THI are presented in Table 1 (Table 1). No statistically significant difference was observed between the groups, ensuring their homogeneity.

**Table 1:** Descriptive data of the sample

	CG		SG		LL	UL	P-value
	MEAN	SD	MEAN	SD			
AGE (Y)	32.10	11.60	39.60	12.38	-18.773	3.773	0.179
EDUCATION	16.30	2.06	16.40	2.67	-2.342	2.142	0.926
PERCEPTION TIME (Y)	4.80	3.33	5.10	3.48	-3.498	2.898	0.846
VAS V PRE	7.10	1.66	8.20	1.62	-2.642	0.442	0.151
VAS D PRE	7.10	2.18	8.20	1.75	-2.959	0.759	0.230
THI PRE	65.80	15.22	61.60	12.43	-8.853	17.253	0.508

**Caption:** N = total number of subjects; SD = standard deviation; (Y) = years; VAS V = Visual Analog Scale for Volume; VAS D = Visual Analog Scale for Discomfort; THI = Tinnitus Handicap Inventory; LL/UL = lower and upper limits of the 95% confidence interval; Statistical test = Independent samples *t*-test

**Figure 2:**
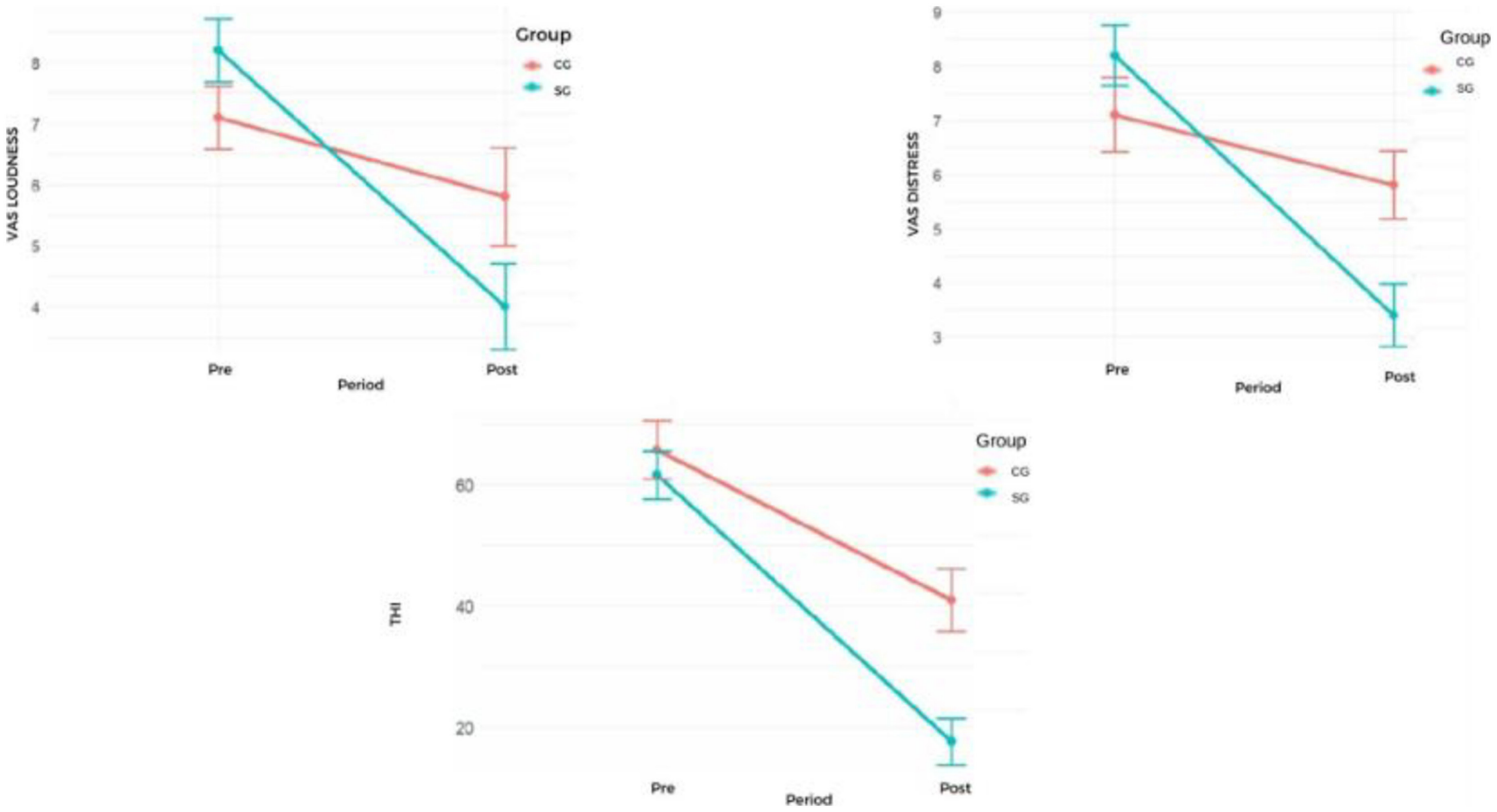
Intra-group comparison of the mean and standard deviation of VAS V, VAS D, and THI

In the intra-group comparison of symptom perception before and after the intervention, a statistically significant difference was observed in both groups for the variables VAS D, VAS V, and THI (Table 2, Figure 2).

**Table 2:** Intra-group comparison for VAS V, VAS D, and THI before and after the intervention

	CG		LL	UL	P-VALUE	SG		LL	UL	P-VALUE
	MEAN	SD				MEAN	SD			
VAS V PRE	7.10	1.66	0.40	2.20	0.009	8.20	1.62	3,14	5,26	< 0.001
VAS V POST	5.80	2.53				4.00	2.21			
VAS D PRE	7.10	2.18	0.62	1.98	0.002	8.20	1.75	3,74	5,86	< 0.001
VAS D POST	5.80	1.99				3.40	1.84			
THI PRE	65.80	15.22	15.65	33.95	< 0.001	61.60	12.43	38,20	49,80	< 0.001
THI POST	41.00	16.50				17.60	12.32			

**Caption:** CG = Control Group; SG = Study Group; VAS V = Visual Analog Scale for Volume; VAS D = Visual Analog Scale for Discomfort; THI = Tinnitus Handicap Inventory; LL/UL = lower and upper limits of the 95% confidence interval; Statistical test = independent samples *t*-test

**Caption:** CG = Control Group; SG = Study Group; VAS = Visual Analog Scale; THI = Tinnitus Handicap Inventory; Pre = pre-intervention; Post = post-intervention

In the comparison of symptom perception between groups at the pre- and post-intervention moments, a statistically significant difference was found for VAS D and the THI questionnaire at the post-intervention moment, with a greater reduction in perceived discomfort and impact on quality of life observed in the SG (Table 3).

**Table 3:** Between-group comparison for VAS V, VAS D, and THI after the intervention

	GROUP	MEAN	SD	LL	UL	P - VALUE
VAS V PRE	CG	7.10	1.66	-2.64	0.44	0.151
	SG	8.20	1.62			
VAS V POST	CG	5.80	2.53	-0.43	4.03	0.107
	SG	4.00	2.21			
VAS D PRE	CG	7.10	2.18	-2.96	0.76	0.230
	SG	8.20	1.75			
VAS D POST	CG	5.80	1.99	0.60	4.20	**0.012**
	SG	3.40	1.84			
THI PRE	CG	65.80	15.22	-8.85	17.25	0.508
	SG	61.80	12.43			
THI POST	CG	41.00	16.50	9.72	37.08	**0.002**
	SG	17.60	12.32			

**Caption:** CG = Control Group; SG = Study Group; VAS V = Visual Analog Scale for Volume; VAS D = Visual Analog Scale for Discomfort; THI = Tinnitus Handicap Inventory; SD = standard deviation; LL/UL = lower and upper limits of the 95% confidence interval; Statistical test = independent samples *t*-test

In the analysis of LLAEP within groups and between ears, no statistically significant difference was observed (p>0.05); therefore, the data were grouped.

In Table 4, the comparison of LLAEP amplitude and latency variables at the pre- and post-intervention moments in the CG can be observed. A statistically significant difference was found for the duration variable of the P3 component.

**Table 4:** Results of the mean, minimum, maximum, difference, and p-value for latency, amplitude, and duration of the LLAEP potentials in the Control Group before and after the intervention

VARIABLE	MEAN	MINIMUM	MAXIMUM	DIF.	LL	UL	P-VALUE
L P1 - PRE	64,85	45	85	3.70	-5,72	13,12	0.397
L P1 - POST	61,15	45	73				
A P1 - PRE	5,25	1,31	10,58	-0.09	-2,28	2,11	0.932
A P1 - POST	5,28	2,06	12,16				
L N1 - PRE	112,95	89	142	3.40	-6,18	12,98	0.443
L N1 - POST	109,55	86	124				
A N1 - PRE	-2,52	-12,68	-3,21	1.43	-3,37	6,23	0.517
A N1 - POST	-3,95	-13,24	-2,01				
L P2 - PRE	186,5	141	239	-7.55	-42,53	27,43	0.637
L P2 - POST	194,05	145	261				
A P2 - PRE	5,46	0,65	10,92	-0.54	-2,66	1,58	0.580
A P2 - POST	6	1,63	11,09				
L N2 - PRE	251,85	167	292	-11.80	-63,87	40,27	0.621
L N2 - POST	263,65	181	350				
A N2 - PRE	-1,27	-7,49	-0,6	-0.70	-2,45	1,04	0.387
A N2 - POST	-0,57	-6,38	0,44				
L P3 - PRE	336,95	280	419	16.30	-8,03	40,63	0.164
L P3 - POST	320,65	230	363				
A P3 - PRE	3,6	0,84	9,11	-0.74	-3,46	1,98	0.555
A P3 - POST	4,34	0,97	11,74				
DURATION P3 - PRE	95,1	60	154	-3.80	-6,69	-0,91	**0.016**
DURATION P3 - POST	98,9	60	170				

**Caption:** Diff = difference; L = latency; A = amplitude; Pre = pre-intervention; Post = post-intervention; LL/UL = lower and upper limits of the 95% confidence interval; Statistical test = paired *t*-test

In Table 5, the comparison of LLAEP amplitude and latency variables at the pre- and post-intervention moments in the SG can be observed. A statistically significant difference was found for the duration variable in the CG and for the duration and amplitude of the P3 component in the SG.

**Table 5:** Results of the mean, minimum, maximum, difference, and p-value for latency, amplitude, and duration of the LLAEP potentials in the Study Group

VARIABLE	MEAN	MINIMUM	MAXIMUM	DIF	LL	US	P-VALUE
L P1 - PRE	64,3	46	83	4.70	-6,62	16,02	0.372
L P1 - POST	59,6	45	77				
A P1 - PRE	4,13	0,34	8,74	-0.96	-4,07	2,16	0.505
A P1 - POST	5,09	0,11	10,23				
L N1 - PRE	111,7	90	145	8.05	-7,37	23,47	0.268
L N1 - POST	103,65	82	132				
A N1 - PRE	-3,45	-13,35	-0,22	1.98	-3,53	7,49	0.438
A N1 - POST	-8,17	-14,82	-1,51				
L P2 - PRE	190,65	161	227	11.10	-3,65	25,85	0.123
L P2 - POST	179,55	128	220				
A P2 - PRE	3,23	0,44	7,08	-1.33	-3,04	0,38	0.112
A P2 - POST	4,56	1,58	8,17				
L N2 - PRE	267,35	214	325	11.80	-14,92	38,52	0.344
L N2 - POST	255,55	203	334				
A N2 - PRE	-1,42	-6,29	-0,1	-0.15	-2,85	2,55	0.905
A N2 - POST	-3,4	-9,46	-0,24				
L P3 - PRE	331,1	264	381	6.40	-18,19	30,99	0.571
L P3 - POST	324,7	284	348				
A P3 - PRE	2,91	0,76	9,57	-1.82	-3,05	-0,58	**0.009**
A P3 - POST	4,72	2,2	9,46				
DURATION P3 - PRE	104,4	55	142	-17.20	-28,34	-6,06	**0.007**
DURATION P3 - POST	121,6	90	186				

**Caption:** Diff = difference; L = latency; A = amplitude; Pre = pre-intervention; Post = post-intervention; LL/UL = lower and upper limits of the 95% confidence interval; Statistical test = paired *t*-test

**Figure 3:**
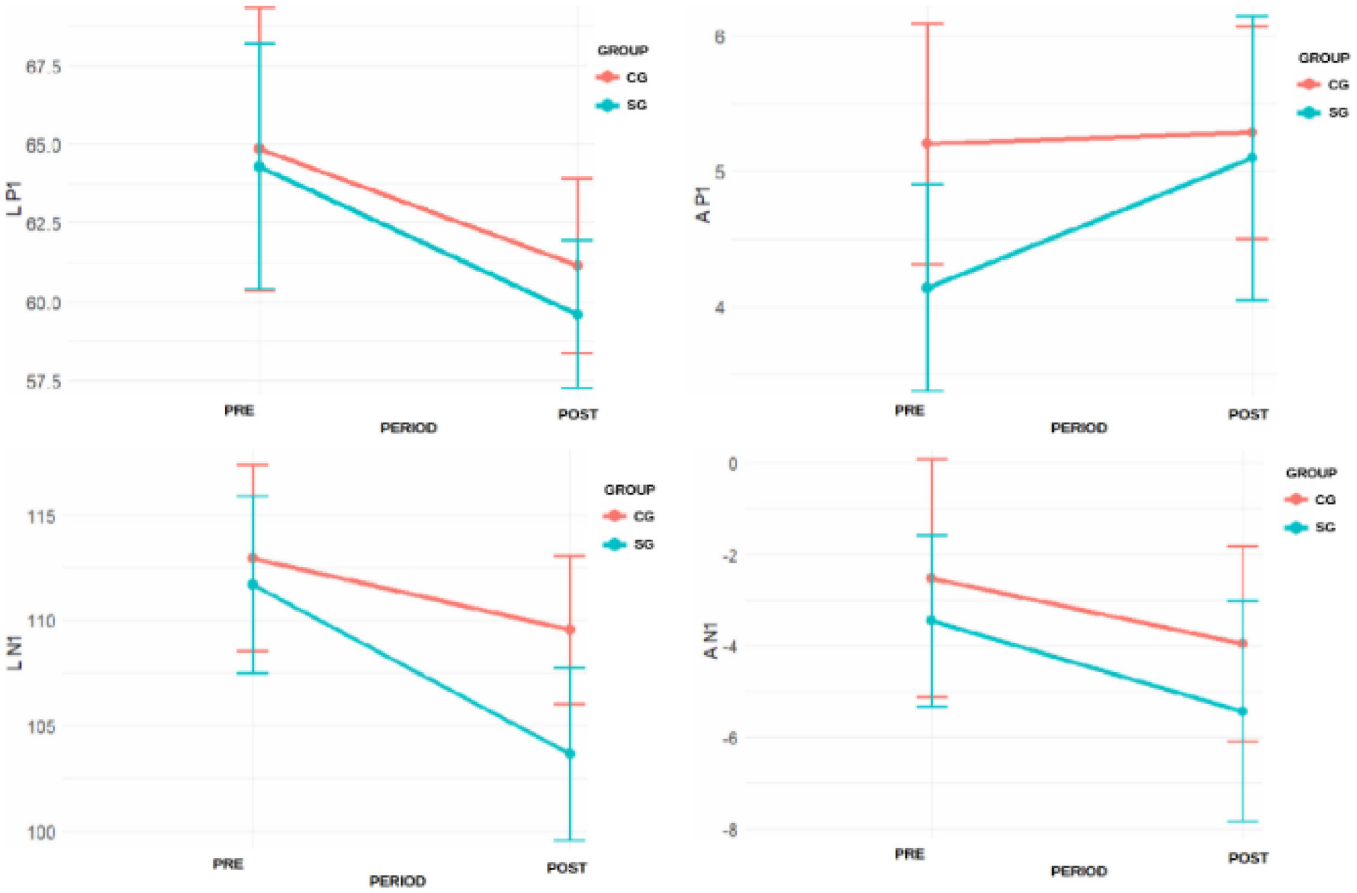
Pre- and post-comparison of latency and amplitude values of the P1 and N1 potentials in both groups

**Figure 4:**
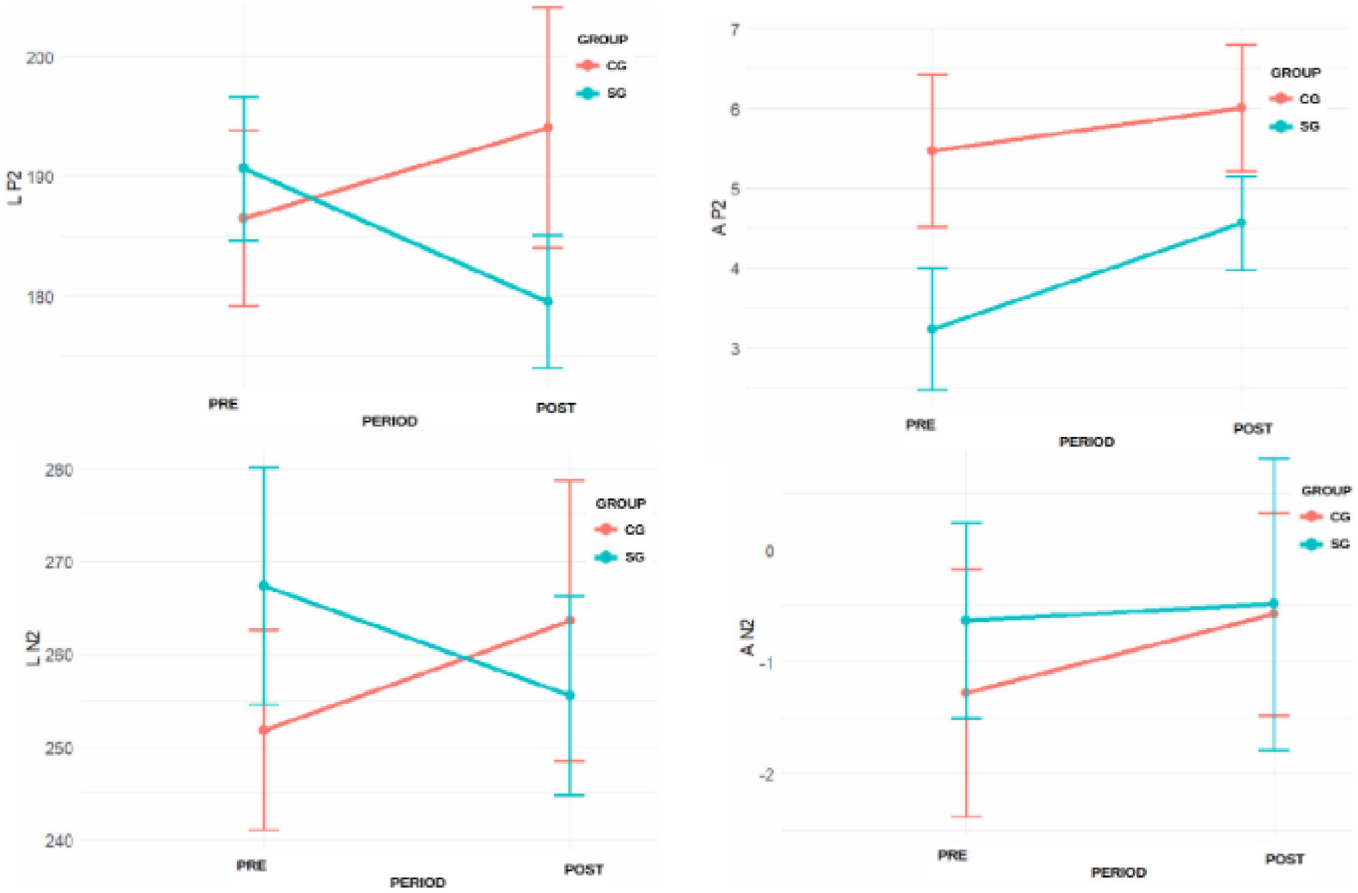
Pre- and post-comparison of latency and amplitude of the P2 and N2 potentials in both groups

**Figure 5:**
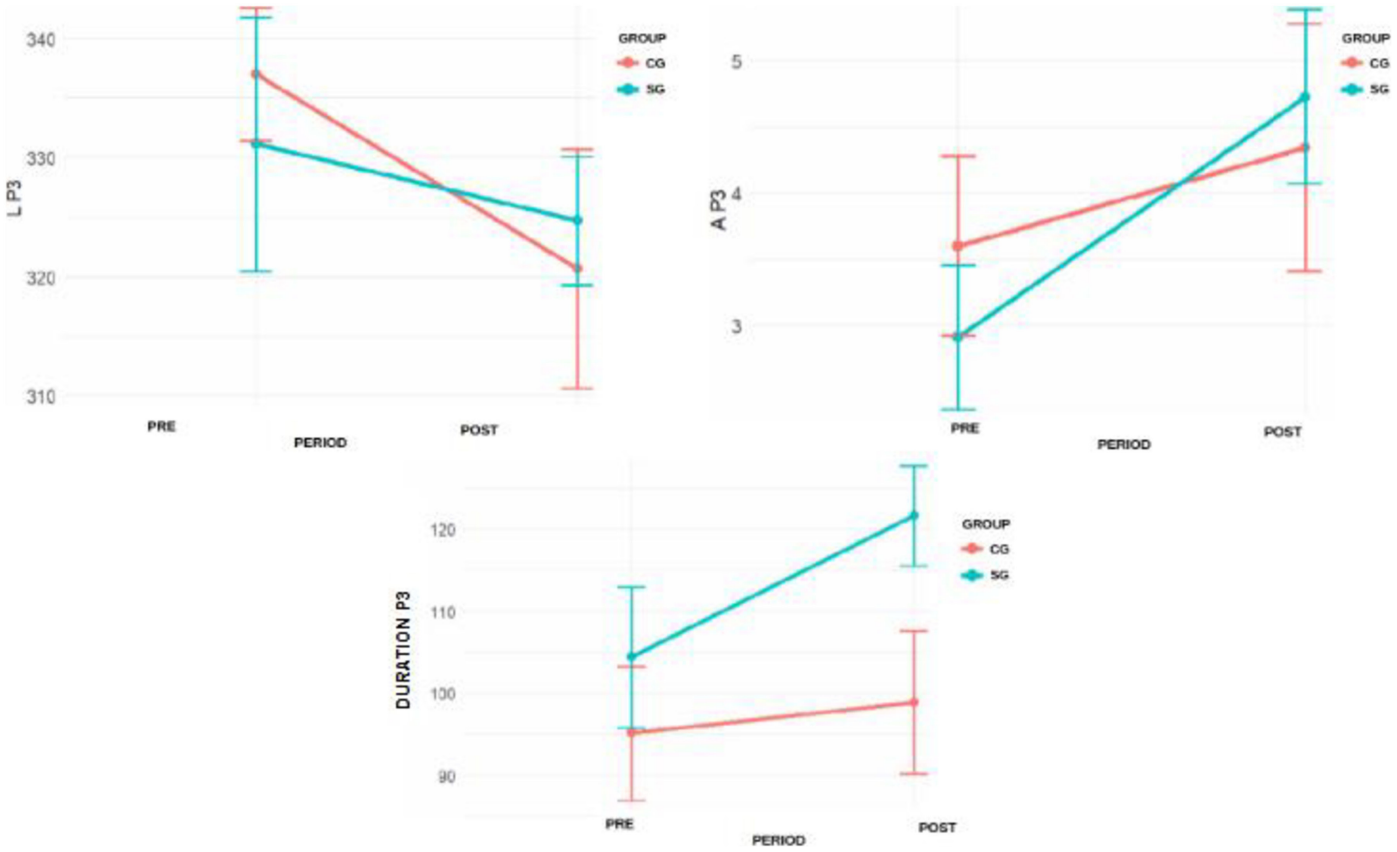
Pre- and post-comparison of latency, amplitude, and duration values of the P3 potential in both groups

The following graphs (Figure 3, Figure 4, and Figure 5) represent the pre- and post-intervention comparison of LLAEP latency, amplitude, and duration in the CG and SG.

**Caption:** CG = Control Group; SG = Study Group; L = latency; A = amplitude; Pre = pre-intervention; Post = post-intervention

**Caption:** CG = Control Group; SG = Study Group; L = latency; A = amplitude; Pre = pre-intervention; Post = post-intervention

**Caption:** CG = Control Group; SG = Study Group; L = latency; A = amplitude; Pre = pre-intervention; Post = post-intervention

**Figure 6:**
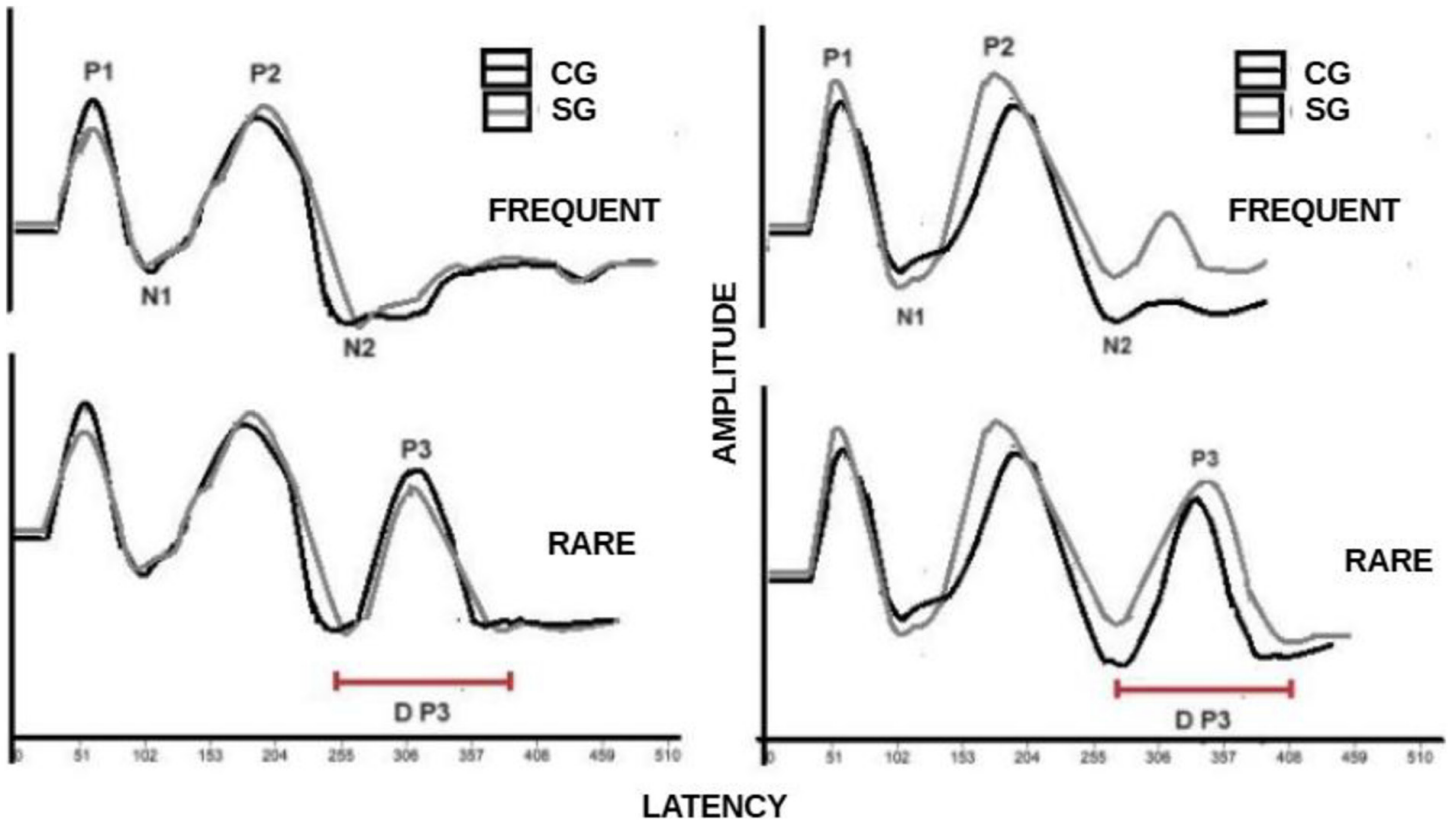
Graphical representation of the grand mean of LLAEP before and after the intervention in the CG and SG

In Figure 6, the graphical representation of the grand average of LLAEP before and after the intervention in both groups can be visualized. The graphical representation of the grand average was generated from the results of the mean values of the potential components.

**Caption:** CG = Control Group; SG = Study Group; D = duration

In the SG, duration and amplitude showed statistically significant changes.

It is noteworthy that, in this study, no adverse events occurred.

## DISCUSSION

The present study investigated the effects of MAT on tinnitus perception and on LLAEP, demonstrating a significant reduction in discomfort and impact on quality of life, in addition to changes compatible with greater neural recruitment and maintenance. These findings highlight MAT as an effective intervention capable of promoting functional adaptations in auditory and cognitive areas through stimuli that favor auditory neuroplasticity^(20)^.

Pre-intervention data demonstrate all that is necessary in studies comparing groups in interventions, highlighting the homogeneity of the data for comparison and providing full reliability for the statistical analyses. In addition, researchers emphasize that randomized, double-blind clinical trials with a control group are considered the gold standard for evaluating treatment efficacy and reveal reliable clinical changes^(21)^.

Statistically significant differences were observed in the pre- and post-intervention comparisons in both groups for the variables VAS D, VAS V, and the THI questionnaire. These analyzed variables refer to subjective response patterns of individuals, and the positive results in both groups may be associated with the welcoming approach to the participants and active listening, which occurred both in the study group and in the group that received passive intervention. However, it should be emphasized that the SG showed a statistically significant and expressive change in relation to THI when compared with the results of the CG, demonstrating the strong influence of MAT on the quality of life of these subjects.

It was also observed that participants obtained benefits from the intervention, expanding the treatment options available for tinnitus by including MAT. Previous studies involving training in the auditory-cognitive modality have also demonstrated positive effects in the management of this symptom^(20,22)^.

These results reinforce the idea that active interventions may have notable effects in several clinical conditions, which explains the improvement in symptom perception in the individuals of the present study.

The improvement of symptoms in the CG may be attributed both to the positive expectation generated by the symbolism of treatment and to the effect of passive stimulation, which involved visual and auditory exposure, favoring tinnitus perception without evoking emotions or negative feelings related to the discomfort caused by the symptom^(23)^. Although participants were blinded during the intervention, that is, they did not know to which group they belonged, the findings are consistent with the literature indicating benefits of this modality due to belief in the effectiveness of a supposed intervention^(24)^. It should be emphasized that the activities of the passive stimulation group did not present organization or hierarchy of auditory abilities, which indicates that the observed improvement did not result from mechanisms of auditory system plasticity, but rather from the welcoming approach and the positive effect provided by the experience.

Furthermore, when comparing the variables of symptom perception through the VAS scale and the THI questionnaire between the pre- and post-intervention moments across groups, a greater reduction in the perception of discomfort, volume, and improvement in the impact on quality of life was observed in the SG. This fact enabled differences between the groups at the post-intervention moment. This finding reveals that MAT was more effective compared to passive intervention. It is therefore suggested that the stimulation of auditory abilities through musical stimuli associated with cognitive stimulation activates regions of the frontal and temporal cortex. In addition, it stimulates regions of the limbic system, encompassing auditory and non-auditory regions affected in subjects suffering from tinnitus disorder^(25)^.

These results corroborate those described by other researchers regarding MAT, who report that it provides benefits in quality of life, depressive symptoms, cognitive aspects, temporal resolution, and limitations in activities of daily living^(26,27)^. MAT is based on musicality, which encompasses mechanisms of musical processing and its connection with biochemical, functional, and physiological processes of the auditory system, including auditory, cognitive, and musical association aspects^(28)^. Therefore, it can be predicted that the satisfactory results in symptom perception and neuroplasticity in the SG are associated with auditory and musical stimulation.

Regarding the measurement of neuroplastic effects, in the comparison of the results of LLAEP components P1, N1, P2, N2, and P3, a statistically significant difference was observed for the duration variable of the P3 component in the CG. In the SG, a statistically significant difference was observed for the duration and amplitude variables of the P3 component, with mean values increasing in both groups after the intervention period proposed for each group.

Amplitude is related to the amount of neural recruitment, whereas duration is related to the maintenance of the neural activity performed^(29)^. P3 is an auditory-cognitive potential, that is, it corresponds to auditory abilities performed and is related to cognitive aspects such as memory and attention. Thus, the results of the present study demonstrate improvement in auditory and attentional aspects in the group that received the intervention through MAT. It is important to highlight that the interpretation of these results considered the morphological analysis of the P3 component (Figure 2), taking into account the joint analysis of the duration and amplitude of the component before and after the intervention.

Although the CG showed a difference in component duration between the pre- and post-intervention moments, there was no significant increase in neural recruitment. This indicates greater maintenance of neural activity (reflected in the longer duration of the P3 component) without an increase in the number of recruited neural fibers. Thus, it is assumed that passive intervention had limited effects on the neural organization of the subjects.

The SG increased the time of neural maintenance along with a considerable increase in the recruitment of auditory neurons. This finding suggests improvement in synaptic activity after the proposed training. The increase in amplitude combined with the duration of P3 in the SG also reflects the individuals’ ability to more easily perceive the verbal stimuli presented during the examination, demonstrating improvement in auditory discrimination aspects and attentional abilities. Auditory stimulation disconnects tinnitus and directs the focus of the Central Auditory Nervous System, relieving the symptom by promoting neural reorganization and reallocating each neural activity to its specific function^(20,22)^.

The amplitude of P3 may be reduced in individuals with tinnitus, suggesting that there may be inhibition in attention to external stimuli in individuals with tinnitus, without modifications in latency^(30)^. In the present study, this difference was not observed for any electrophysiological variable in the comparison between groups before the intervention, which ensured sample homogeneity and reinforces that the electrophysiological results are indeed due to the intervention.

This study did not reveal statistically significant differences in the latency and amplitude of the P1, N1, P2, and N2 components. This finding may be explained by the role of tinnitus as an additional stimulus that affects all LLAEP components. The continuous emission of afferent signals generated subcortically by tinnitus appears to provoke an adaptation in the processing of auditory signals in general. This adaptation suggests a compensatory brain mechanism that may influence LLAEP components in individuals with tinnitus, since these potentials are related both to the activity of auditory pathways and to neuronal activity. During the application of the Oddball paradigm, it is possible that individuals with tinnitus simultaneously perceive a frequent stimulus, the continuous tinnitus stimulus, and a rare stimulus, and this interaction of stimuli may hinder concentration on the rare stimulus, generating changes in the latency and amplitude of long-latency potential components^(31)^.

In general, the present research demonstrates that MAT promotes positive changes in the neuroplasticity of the auditory system and changes in symptom perception. The application of MAT provides novelty, since there are no studies in the literature addressing neuroplasticity results, visualized through LLAEP, together with symptom perception in subjects with tinnitus disorder.

The longitudinal design presented challenges, such as identifying subjects who met the inclusion criteria for intervention within a short period of time and offering passive intervention followed by MAT to all subjects in the CG. Although the study included the number of subjects established in the sample size calculation, it should be noted that a larger sample would be appropriate to verify possible effects on the other LLAEP components, since it would increase statistical power. This aspect may be considered a limitation of the study. Other possible limitations include the possibility of selection bias, the short intervention period, since it is believed that a greater number of sessions could impact better results, and the absence of long-term follow-up evaluations.

Despite this, the results stimulated the researchers’ interest in continuing the study, considering the difficulties and limitations mentioned. For future research, audiological monitoring of individuals with tinnitus disorder is recommended in order to evaluate the long-term benefits of the intervention.

This study provides relevant evidence for science and clinical speech-language and hearing practice in the management of tinnitus, demonstrating that MAT is a validated and established technique to be applied. Furthermore, it offers a non-invasive and promising approach for the management of this disorder and may be incorporated as a complementary strategy in tinnitus management in clinical contexts, although further studies are needed to confirm its long-term effectiveness.

## CONCLUSION

Through this clinical trial, it can be concluded that Musical Auditory Training (MAT) promoted positive changes in the neuroplasticity of the auditory system compared to passive control. In addition, a reduction in the perception and volume of tinnitus disorder was observed in the participating adults, reflecting a significant improvement in the subjects’ quality of life.

Regarding the variables studied, MAT promoted improvement in the duration and amplitude of the auditory-cognitive component P3, whereas passive intervention produced positive changes only in the amplitude of P3, demonstrating the greater benefit of active intervention.

Despite the limitations of the study, this article demonstrates that MAT is an effective intervention available for implementation in clinical practice, offering speech-language and hearing professionals an innovative and evidence-based strategy to reduce tinnitus and improve the auditory-cognitive function of individuals suffering from this symptom.
