# Supplementary material for: Tinnitus disorder and musical auditory training: a double-blind randomized controlled clinical trial
Source: Codas. 2026 Jun 15;38(4):e20250150. doi: 10.1590/2317-1782/e20250150en (PMC13322486; doi:10.1590/2317-1782/e20250150en)
Supplement: Translation

# Translation: Transtorno do zumbido e treinamento auditivo musical: um ensaio clínico duplo cego randomizado controlado

**DOI:** 10.1590/2317-1782/e20250150pt

**Authors:** Christine Grellmann Schumacher, Katya Guglielmi Marcondes Freire, Denis Altieri de Oliveira Moraes, Michele Vargas Garcia, Dayane Domeneghini Didoné

**Affiliations:** 1 Departamento de Fonoaudiologia, Centro de Ciências da Saúde, Universidade Federal de Santa Maria – UFSM - Santa Maria (RS), Brasil.

**Keywords:** Zumbido, Percepção Auditiva, Audição, Ensaio Clínico Controlado, Potenciais Evocados Auditivos

## Abstract

**Objetivo:**

Verificar o efeito do Treinamento Auditivo Musical (TAM) na neuroplasticidade do sistema auditivo e na percepção do zumbido em adultos.

**Método:**

Ensaio clínico randomizado duplo-cego, aprovado pelo Comitê de Ética em Pesquisa. Foram incluídos vinte adultos com queixa de zumbido há pelo menos seis meses, intensidade maior que quatro pontos na Escala Visual Analógica (EVA), limiares auditivos normais bilateralmente e sincronia neural preservada em tronco encefálico. Os participantes foram distribuídos aleatoriamente em dois grupos: Controle (GC, n=10), submetido à estimulação passiva com material audiovisual sem tarefas, e Estudo (GE, n=10), submetido ao TAM. Ambos realizaram oito sessões, duas vezes por semana, ao longo de quatro semanas. As avaliações compreenderam Potenciais Evocados Auditivos de Longa Latência (PEALL) verbais, para investigar neuroplasticidade, além da EVA (volume e incômodo) e do Tinnitus Handicap Inventory (THI), para mensurar a percepção do sintoma.

**Resultados:**

Ambos os grupos apresentaram melhora na percepção do zumbido após a intervenção. Contudo, a análise diferenciada mostrou que o GC apresentou mudanças restritas, com melhora apenas na duração do componente P3 dos PEALL. Já o GE apresentou alterações mais amplas, incluindo mudanças significativas na amplitude e duração do P3, indicando maior recrutamento neural associado ao processamento auditivo. Além disso, apenas o GE demonstrou reduções consistentes no volume e no incômodo do zumbido, bem como no impacto sobre a qualidade de vida, refletido nas pontuações do THI.

**Conclusão:**

Após quatro semanas, o TAM mostrou-se superior à estimulação passiva, promovendo alterações positivas na neuroplasticidade auditiva, diminuição mais expressiva da intensidade e do incômodo do zumbido e melhora significativa na qualidade de vida dos participantes.

## INTRODUÇÃO

O transtorno do zumbido é um sintoma multifacetado e originado por diversos mecanismos. Em 2021, a literatura científica propôs uma diferenciação conceitual entre o termo “zumbido” e “transtorno do zumbido”, sendo que o zumbido se limita à percepção auditiva isolada, e o transtorno do zumbido inclui aspectos emocionais, cognitivos e comportamentais, frequentemente associados a sofrimento psíquico, desordens funcionais e alterações no comportamento^(1)^.

A literatura indica que o transtorno do zumbido não está restrito a disfunções periféricas do sistema auditivo, mas envolve alterações centrais que refletem em mudanças na neuroplasticidade do sistema auditivo, gerando uma desorganização nas vias auditivas centrais. Essas alterações impactam significativamente a organização das vias auditivas, modificando o processamento neural^(2)^.

Entre as hipóteses etiológicas mais aceitas, destaca-se o papel das mudanças neuroplásticas centrais. Indivíduos com zumbido apresentam aumento na atividade das vias auditivas e comprometimento em áreas corticais ligadas a funções cognitivas e emocionais, como os sistemas límbico e cognitivo^(3)^. Nesse contexto, estratégias como o treinamento auditivo têm sido investigadas, considerando sua capacidade de promover reorganização neural por meio da estimulação acústica e de estratégias cognitivas e metacognitivas^(4)^.

Sendo assim, o treinamento auditivo musical emerge como uma abordagem potencialmente eficaz. Esse treinamento estimula não apenas as habilidades auditivas, mas também funções cognitivas, como a atenção e a memória operacional^(5,6)^.

Entre as ferramentas de avaliação dos efeitos de intervenção auditiva estão os Potenciais Evocados Auditivos de Longa Latência (PEALL) se destacam por fornecerem informações detalhadas sobre a atividade do Sistema Nervoso Auditivo Central, incluindo habilidades de discriminação e memória auditiva^(7)^. Alterações nesses potenciais, frequentemente observadas em indivíduos com transtorno do zumbido, refletem reorganizações em redes neurais subcorticais, temporoparietais, pré-frontais e límbicas, comprometendo tanto funções auditivas quanto cognitivas^(8)^.

Embora o treinamento auditivo seja apontado como uma estratégia promissora para aprimorar a plasticidade neural em indivíduos com transtorno do zumbido, a literatura carece de investigações que explorem sua eficácia. Em particular, a aplicação do treinamento auditivo musical nesse contexto ainda não foi amplamente estudada.

Diante dessa lacuna, este estudo se fundamenta na hipótese de que o treinamento auditivo musical pode promover a reorganização das vias auditivas centrais em indivíduos com transtorno do zumbido, resultando em alterações neurais favoráveis e melhora na percepção do sintoma. Assim, o objetivo deste estudo foi avaliar os efeitos do TAM em adultos com transtorno do zumbido, considerando tanto os aspectos funcionais do processamento auditivo central quanto as repercussões clínicas do sintoma. O caráter inovador desta investigação está na utilização conjunta do TAM e dos PEALL, permitindo compreender de forma integrada tanto os aspectos subjetivos da percepção do sintoma quanto os correlatos objetivos da atividade neural. Ao explorar simultaneamente essas dimensões, este estudo contribui com evidências inovadoras para o manejo clínico do zumbido, oferecendo uma intervenção não invasiva, de baixo custo e com potencial de aplicação ampla na prática fonoaudiológica.

## MÉTODO

Ensaio clínico randomizado duplo cego, controlado, registrado na plataforma *Clinical Trials* (NCT06371287), baseado no Consort checklist, aprovado pelo Comitê de Ética e Pesquisa sob número 64696022.1.0000.5346. Todos os procedimentos foram realizados no ambulatório de audiologia e eletrofisiologia da audição do Serviço de Atendimento Fonoaudiológico da Universidade Federal de Santa Maria. Todos os indivíduos envolvidos assinaram o Termo de Consentimento Livre e Esclarecido.

O recrutamento dos participantes ocorreu entre abril de 2023 e fevereiro de 2024. O período de intervenção teve duração de quatro semanas, com reavaliação realizada após quatro semanas do término das sessões.

O estudo caracteriza-se por dois grupos, um que recebeu intervenção por meio do Treinamento Auditivo Musical e outro, grupo controle, com intervenção passiva. A distribuição dos sujeitos ocorreu de forma aleatória, na qual foram randomizados entre os dois grupos. A randomização foi realizada por um pesquisador independente que alocou os sujeitos em uma planilha do *excel* em ordem alfabética e realizou o sorteio da intervenção pela ordem nominal, ou seja, o sorteio foi realizado para cada sujeito alocado na planilha. A ocultação da alocação foi assegurada, uma vez que o pesquisador independente somente divulgou a designação dos grupos aos responsáveis pela intervenção após a conclusão das avaliações iniciais. O estudo caracterizou-se como duplo cego, uma vez que os participantes não tinham conhecimento do tipo de intervenção recebida. Além disso, os pesquisadores responsáveis pelas avaliações iniciais e finais, bem como os juízes que analisaram os registros eletrofisiológicos, permaneceram cegos em relação à alocação dos grupos. Apenas o pesquisador encarregado da aplicação das intervenções tinha acesso à designação, não participando das etapas de avaliação nem da análise dos dados.

Considerando o cálculo amostral para verificar a diferença do escore médio entre as duas avaliações, antes e depois em dados pareados por sujeito, foram estabelecidos os seguintes parâmetros: tamanho do efeito igual a 0.8 desvios, nível de significância nominal (probabilidade de erro do tipo I) igual à 5% e poder do teste de 75%. Dessa forma, o número mínimo de indivíduos em cada grupo deveria ser de pelo menos 10 indivíduos.

Foram estabelecidos os seguintes critérios de inclusão:

- Indivíduos de ambos os sexos da faixa etária entre 18 anos e 55 anos;
- Queixa de transtorno do zumbido;
- Nota de incômodo de no mínimo quatro na Escala Visual Analógica, considerado um incômodo moderado do sintoma, caracterizando o transtorno do zumbido;
- Percepção mínima de seis meses do transtorno do zumbido em ambas as orelhas^(9)^;
- Consulta prévia com médico otorrinolaringologista;
- Limiares auditivos dentro dos padrões de normalidade bilateralmente ou perda auditiva sensorioneural de grau até leve na média quadritonal (500, 1000, 2000 e 4000Hz)^(10)^;
- Curva timpanométrica do tipo A bilateralmente;
- Normalidade no Potencial Evocado Auditivo de Tronco Encefálico (PEATE)^(11)^, indicando integridade do sistema auditivo;
- Ter normalidade no Mini Exame do Estado Mental^(12)^.

Já os critérios de exclusão foram:

- Alterações de fala, doenças psiquiátricas ou neurológicas diagnosticadas ou aparentes;
- Histórico de trauma craniano ou cerebral;
- Presença de modulação para zumbido somatossensorial e vascular, detectados por meio da Triagem do zumbido de origem vascular^(13)^ e do Teste de modulação somatossensorial do zumbido^(14)^;
- Apresentar sintomas e/ou diagnóstico de comprometimento de orelha externa/média^(15)^;
- Ter iniciado novo tratamento (farmacológico ou terapêutico) ou ter sido diagnosticado com alguma doença de qualquer origem no último mês.
- Estar realizando outra intervenção para o zumbido durante a pesquisa;
- Pontuação maior do que 11 pontos na Escala Hospitalar de Ansiedade e Depressão (EHAD), indicando risco moderado de ansiedade ou depressão^(16)^.

Os procedimentos escolhidos para esse estudo foram definidos segundo a composição amostral. Estes foram realizados em dois momentos pelo mesmo examinador. Em um primeiro momento o sujeito compareceu para realizar os procedimentos de avaliação do zumbido, questionários e avaliação audiológica básica (uma hora de duração), e em outro momento para a avaliação eletrofisiológica (uma hora de duração). A ordem dos procedimentos de avaliação foi a seguinte: anamnese, inspeção visual do meato acústico externo, audiometria tonal liminar, logoaudiometria, imitanciometria e aplicação dos questionários *Tinnitus handicap inventory (THI)* e Escala visual analógica (EVA) . Para avaliação eletrofisiológica, inicialmente realizou-se o Potencial Evocado Auditivo de Tronco o Encefálico (PEATE) para verificar a integridade da via auditiva e posteriormente o Potencial Evocado Auditivo de Longa Latência (PEALL). Após, os sujeitos selecionados foram submetidos à intervenção (TAM ou estimulação passiva) e posteriormente à reavaliação por meio dos PEALL para analisar o desfecho das intervenções aplicadas.

### Potencial Evocado Auditivo de Longa Latência (PEALL)

Os Potenciais Evocados Auditivos de Longa Latência (PEALL) foram utilizados para avaliar a neuroplasticidade antes e após quatro semanas de intervenção. As medidas foram realizadas com o *SmartEP* (Intelligent Hearing Systems), utilizando fones de inserção e seguindo protocolos para minimizar artefatos. A preparação incluiu limpeza da pele com pasta abrasiva (*NUPREP*) e fixação dos eletrodos (eletrodo ativo em Cz, terra em Fpz e os eletrodos referência foram posicionados nos lóbulos das orelhas esquerda (A1) e direita (A2) com pasta eletrolítica e esparadrapo. Os registros foram obtidos com um paradigma *oddball* (/ba/ e /di/), em polaridade alternada, intensidade de 80 dBnNA e 300 estímulos (240 BA e 60 DI). Foram seguidos critérios rigorosos para controle de artefatos e parâmetros, como taxa de 1,10/s, janela de registro de 510ms, ganho de 100K, filtro de 100-3000Hz, janela do eletroencefalograma (EEG) de 31%, impedância de no máximo 3KΩ. Os artefatos foram monitorados, para não ultrapassarem 10% do número de estimulações, mantendo a relação sinal-ruído ≥ 1dB e o ruído residual ≤ 0,11dB.

Dois juízes especialistas cegados analisaram a latência das ondas P1, N1, P2, N2 e P300^(17)^. Não houve divergência nas marcações. Ressalta-se que a amplitude foi considerada de acordo com a morfologia dos componentes, sem considerar parâmetros mínimos de referência, visto que o objetivo do estudo foi mensurar a atividade neuroplástica. A padronização garantiu a confiabilidade dos resultados. Após a marcação dos componentes do PEALL pelos juízes especialistas, foi calculada a duração do componente P3, considerando o início e o final da onda. O início da onda foi considerado no ponto inicial do vale e o final no término do vale da onda. A duração foi calculada a partir da subtração do valor em milissegundos do final e do início da onda P3. O cálculo foi realizado por meio de aplicação de fórmula de subtração em planilhas do *excel.*

### Tinnitus handicap inventory (THI)

O *Tinnitus Handicap Inventory* (THI) foi utilizado para medir a influência do transtorno do zumbido na qualidade de vida dos participantes e classificá-los em níveis de gravidade com base na pontuação total^(18)^. Composto por 25 questões, o questionário avalia a percepção do zumbido e seu impacto funcional, emocional e catastrófico. Os participantes responderam às questões escolhendo entre “sim”, “não” ou “às vezes”, de acordo com a relevância para sua experiência. A pontuação obtida permitiu a classificação em cinco graus: ligeiro, leve, moderado, severo e catastrófico. O THI foi aplicado tanto na avaliação inicial quanto na reavaliação, realizada ao final de quatro semanas de intervenção, para analisar mudanças no impacto percebido.

### Escala visual analógica (EVA)

A Escala Visual Analógica (EVA) é amplamente utilizada para mensurar a dor crônica e, em indivíduos com transtorno do zumbido, avalia o grau de incômodo causado pelo sintoma^(19)^. Trata-se de uma medida subjetiva na qual os participantes atribuem uma nota de 0 a 10, considerando tanto o incômodo (EVA I) quanto ao volume (EVA V) percebida do zumbido. Para caracterização do transtorno, foram incluídos apenas sujeitos com pontuação na EVA maior ou igual a 4^(19)^. Além disso, a EVA foi aplicada antes e após a intervenção, realizada durante quatro semanas, para monitorar mudanças na percepção do sintoma.

### Procedimentos de intervenção

#### Grupo Estudo - Treinamento Auditivo Musical

O treinamento musical baseou-se no protocolo proposto por Freire et al.^(6)^, originalmente desenvolvido para idosos com próteses auditivas, adaptado neste estudo como intervenção para zumbido. O protocolo visa o treinamento auditivo por meio da hierarquização das habilidades auditivas associadas à musicalidade.

Foram realizadas oito sessões, distribuídas em quatro semanas, com duas sessões semanais de 40 a 50 minutos. Os sujeitos utilizaram fones supra-aurais calibrados para o nível de conforto auditivo individual no início de cada sessão. As atividades treinaram habilidades como figura-fundo para sons instrumentais, escuta direcionada, duração, frequência, ritmo, fechamento auditivo e memória audiovisual, com ênfase em processamento temporal, memória operacional e atenção seletiva, organizadas em níveis crescentes de dificuldade.

Cada sessão incluiu 10 exercícios por nível, ajustando a complexidade conforme o tempo e a natureza das atividades. Atividades monoaurais iniciaram pela orelha esquerda, considerando a predominância do hemisfério direito no processamento de estímulos não-verbais. O protocolo, disponível online, foi autoexplicativo, com acompanhamento presencial das pesquisadoras, e incluiu instruções claras sobre o objetivo de cada tarefa, adaptando o tempo de resposta para promover a estimulação da memória de trabalho.

Ressalta-se que as pesquisadoras tiveram acesso à versão completa do TAM e aos dados de percentual de acertos de cada sessão, disponíveis no site. O acesso foi autorizado e liberado pela autora responsável.

#### Grupo Controle - intervenção passiva

Para minimizar vieses e avaliar a eficácia do Treinamento Auditivo Musical (TAM) na neuroplasticidade do sistema auditivo e na percepção do transtorno do zumbido, foi incluído um grupo com intervenção passiva. Esse grupo realizou oito sessões ao longo de quatro semanas, com duas sessões semanais, em condições idênticas às do grupo estudo (40 a 50 minutos por sessão), acompanhados das pesquisadoras.

A intervenção passiva utilizou música instrumental (*Sonata para Dois Pianos em Ré Maior, K448*, de Mozart) em conjunto com a exibição de filmes sem som, proporcionando estimulação visual e auditiva. Os filmes selecionados incluíram obras como *Cirque Du Soleil: A Jornada do Homem* e filmes de Chaplin (*Tempos Modernos*, *O Grande Ditador*, *Em Busca do Ouro* e *Luzes da Ribalta*), exibidos em ordem aleatória^(6)^.

Essa abordagem buscou avaliar o impacto da exposição musical sem a estruturação de habilidades auditivas, em comparação ao TAM. O volume da música foi ajustado individualmente para não mascarar o zumbido.

## CASUÍSTICA

A casuística foi composta por 20 sujeitos com transtorno do zumbido, sendo 10 no Grupo Controle e 10 no Grupo Estudo. Neste estudo 70 sujeitos entraram em contato para participar da pesquisa, sendo que apenas 20 se adequaram aos critérios da pesquisa. Os outros 50 sujeitos foram excluídos com base em critérios específicos, incluindo dez por pontuação maior que 11 na EHAD, oito pela idade, nove por apresentarem perda auditiva maior do que o grau leve, sete por doenças psiquiátricas ou neurológicas, sete por comprometimento de orelha média, quatro por nota EVA menor que quatro pontos e cinco por presença de modulação nos testes de triagem do componente somatossensorial e/ou vascular. Ressalta-se que não houveram eventuais dados ausentes ou perdas de segmentos durante a realização da pesquisa longitudinal.

O fluxograma da casuística do estudo pode ser visualizado na Figura 1

**Figura 1:** Fluxograma

**Legenda:** n: total de sujeitos; EHAD: Escala Hospitalar de Ansiedade e Depressão; EVA: Escala Visual Analógica; GC: Grupo Controle; GE: Grupo Estudo

O desfecho primário do estudo foi a percepção do zumbido e a reorganização das vias auditivas centrais após a aplicação do TAM. Os desfechos secundários incluíram a mensuração do volume e incômodo do zumbido, assim como a latência, amplitude e duração dos PEALL após as intervenções aplicadas.

Os resultados foram tabulados no programa *Microsoft Office Excel* para análise. Foi utilizado o nível de significância de 5% e as análises foram realizadas com o auxílio do software *R.*

Os dados foram testados quanto à normalidade por meio do teste Shapiro-Wilk, enquanto o teste de Levene foi utilizado para avaliar a homogeneidade de variâncias entre os grupos. As variáveis da EVA, THI e de latência, amplitude e duração dos PEALL foram comparadas entre orelha direita e orelha esquerda, entre grupos por meio do teste T para amostras independentes, nos momentos pré e pós intervenção.

Quando analisados os grupos pré e pós, a análise estatística foi realizada por meio do teste T para amostras emparelhadas. Para as análises posteriores, considerou-se a média entre a orelha direita e a orelha esquerda, já que não houve diferença estatisticamente significante entre os lados avaliados, em ambos os grupos.

## RESULTADOS

Foram incluídos seis participantes do sexo feminino e quatro do sexo masculino no GC (n=10). Já no GE foram incluídos cinco sujeitos do sexo feminino e cinco do sexo masculino (n=10). A comparação entre os grupos não evidenciou diferença estatisticamente significante (p-valor=0,653).

Os dados referentes à idade, escolaridade, tempo de percepção, EVA V pré intervenção, EVA I pré intervenção e THI pré intervenção estão apresentados na Tabela 1. Não foi evidenciada diferença estatisticamente significante entre os grupos, garantindo a homogeneidade dos mesmos.

**Tabela 1:** Dados descritivos da amostra

	GC		GE		Li	Ls	P-valor
	MÉDIA	DP	MÉDIA	DP			
IDADE (A)	32.10	11.60	39.60	12.38	-18.773	3.773	0.179
ESCOLARIDADE	16.30	2.06	16.40	2.67	-2.342	2.142	0.926
TEMPO DE PERCEPÇÃO (A)	4.80	3.33	5.10	3.48	-3.498	2.898	0.846
EVA V PRÉ	7.10	1.66	8.20	1.62	-2.642	0.442	0.151
EVA I PRÉ	7.10	2.18	8.20	1.75	-2.959	0.759	0.230
THI PRÉ	65.80	15.22	61.60	12.43	-8.853	17.253	0.508

**Legenda:** N = total de sujeitos; DP = desvio padrão; (A) = anos; EVA V = Escala Visual Analógica para Volume; EVA I = Escala Visual Analógica para Incômodo; THI = Tinnitus Handicap Inventory; Li/Ls = limites do intervalo com 95% de confiança; Teste estatístico = Teste T para amostras independentes

Na comparação da percepção do sintoma intra os grupos pré e pós intervenção, observou-se diferença estatisticamente significante em ambos os grupos para as variáveis EVA I, EVA V e THI (Tabela 2, Figura 2).

**Tabela 2:** Comparação intra grupos para EVA V, EVA I e THI pré e pós intervenção

	GC		Li	Ls	GE			Li	Ls	P-VALOR
	MÉDIA	DP			P-VALOR	MÉDIA	DP			
EVA V PRÉ	7.10	1.66	0.40	2.20	0.009	8.20	1.62	3,14	5,26	< 0.001
EVA V PÓS	5.80	2.53				4.00	2.21			
EVA I PRÉ	7.10	2.18	0.62	1.98	0.002	8.20	1.75	3,74	5,86	< 0.001
EVA I PÓS	5.80	1.99				3.40	1.84			
THI PRÉ	65.80	15.22	15.65	33.95	< 0.001	61.60	12.43	38,20	49,80	< 0.001
THI PÓS	41.00	16.50				17.60	12.32			

**Legenda:** GC = Grupo controle; GE = Grupo estudo; EVA V = Escala Visual Analógica para Volume; EVA I = Escala Visual Analógica para Incômodo; THI = Tinnitus Handicap Inventory; Li/Ls = limites do intervalo com 95% de confiança; Teste estatístico = teste T para amostras independentes

**Figura 2:** Comparação da média e desvio padrão da EVA V, EVA I e THI intra grupos

**Legenda:** GC = Grupo controle; GE = Grupo estudo; EVA = Escala Visual Analógica; THI = Tinnitus Handicap Inventory; Pré = Pré intervenção; Pós = Pós intervenção

Na comparação da percepção do sintoma entre os grupos no momento pré e pós intervenção, constatou-se diferença estatisticamente significante da EVA I e do questionário THI no momento pós intervenção, sendo evidenciado redução maior da percepção de incômodo e impacto na qualidade de vida no GE (Tabela 3).

**Tabela 3:** Comparação entre grupos para EVA V, EVA I e THI pós intervenção

	GRUPO	MÉDIA	DP	**Li**	**Ls**	P - VALOR
EVA V PRE	GC	7.10	1.66	-2.64	0.44	0.151
	GE	8.20	1.62			
EVA V PÓS	GC	5.80	2.53	-0.43	4.03	0.107
	GE	4.00	2.21			
EVA I PRÉ	GC	7.10	2.18	-2.96	0.76	0.230
	GE	8.20	1.75			
EVA I PÓS	GC	5.80	1.99	0.60	4.20	**0.012**
	GE	3.40	1.84			
THI PRÉ	GC	65.80	15.22	-8.85	17.25	0.508
	GE	61.80	12.43			
THI PÓS	GC	41.00	16.50	9.72	37.08	**0.002**
	GE	17.60	12.32			

**Legenda:** GC = Grupo controle; GE = Grupo estudo; EVA V = Escala Visual Analógica para Volume; EVA I = Escala Visual Analógica para Incômodo; THI = Tinnitus Handicap Inventory; DP = desvio padrão; Li/Ls = limites do intervalo com 95% de confiança; Teste estatístico = Teste T para amostras independentes

Na análise dos PEALL intra grupos e entre orelhas não foi observada diferença estatisticamente significante (p>0,05), sendo assim os dados foram agrupados.

Na Tabela 4, é possível observar a comparação das variáveis de amplitude e latência dos PEALL nos momentos pré e pós do GC. Constatou-se diferença estatisticamente significante para a variável duração do componente P3.

**Tabela 4:** Resultados da média, mínimo, máximo, diferença e p-valor para latência, amplitude e duração dos potencial do PEALL no Grupo Controle pré e pós intervenção

VARIÁVEL	MÉDIA	MÍNIMO	MÁXIMO	Dif.	Li	Ls	P-valor
L P1 - Pré	64,85	45	85	3.70	-5,72	13,12	0.397
L P1 - Pós	61,15	45	73				
A P1 - Pré	5,25	1,31	10,58	-0.09	-2,28	2,11	0.932
A P1 - Pós	5,28	2,06	12,16				
L N1 - Pré	112,95	89	142	3.40	-6,18	12,98	0.443
L N1 - Pós	109,55	86	124				
A N1 - Pré	-2,52	-12,68	-3,21	1.43	-3,37	6,23	0.517
A N1 - Pós	-3,95	-13,24	-2,01				
L P2 - Pré	186,5	141	239	-7.55	-42,53	27,43	0.637
L P2 - Pós	194,05	145	261				
A P2 - Pré	5,46	0,65	10,92	-0.54	-2,66	1,58	0.580
A P2 - Pós	6	1,63	11,09				
L N2 - Pré	251,85	167	292	-11.80	-63,87	40,27	0.621
L N2 - Pós	263,65	181	350				
A N2 - Pré	-1,27	-7,49	-0,6	-0.70	-2,45	1,04	0.387
A N2 - Pós	-0,57	-6,38	0,44				
L P3 - Pré	336,95	280	419	16.30	-8,03	40,63	0.164
L P3 - Pós	320,65	230	363				
A P3 - Pré	3,6	0,84	9,11	-0.74	-3,46	1,98	0.555
A P3 - Pós	4,34	0,97	11,74				
DURAÇÃO P3 - Pré	95,1	60	154	-3.80	-6,69	-0,91	**0.016**
DURAÇÃO P3 - Pós	98,9	60	170				

**Legenda:** Dif = diferença; L = Latência; A = Amplitude; Pré = Pré intervenção; Pós = Pós intervenção; Li/Ls = limites do intervalo com 95% de confiança; Teste estatístico = Teste t para amostras emparelhadas

Na Tabela 5 é possível observar a comparação das variáveis de amplitude e latência dos PEALL nos momentos pré e pós intervenção do GE. Constatou-se diferença estatisticamente significante para a variável duração no GC e duração e amplitude do componente P3 no GE.

**Tabela 5:** Resultados da média, mínimo, máximo, diferença e p-valor para latência, amplitude e duração dos potencial do PEALL no Grupo Estudo

VARIÁVEL	MÉDIA	MÍNIMO	MÁXIMO	Dif.	Li	Ls	P-valor
L P1 - Pré	64,3	46	83	4.70	-6,62	16,02	0.372
L P1 - Pós	59,6	45	77				
A P1 - Pré	4,13	0,34	8,74	-0.96	-4,07	2,16	0.505
A P1 - Pós	5,09	0,11	10,23				
L N1 - Pré	111,7	90	145	8.05	-7,37	23,47	0.268
L N1 - Pós	103,65	82	132				
A N1 - Pré	-3,45	-13,35	-0,22	1.98	-3,53	7,49	0.438
A N1 - Pós	-8,17	-14,82	-1,51				
L P2 - Pré	190,65	161	227	11.10	-3,65	25,85	0.123
L P2 - Pós	179,55	128	220				
A P2 - Pré	3,23	0,44	7,08	-1.33	-3,04	0,38	0.112
A P2 - Pós	4,56	1,58	8,17				
L N2 - Pré	267,35	214	325	11.80	-14,92	38,52	0.344
L N2 - Pós	255,55	203	334				
A N2 - Pré	-1,42	-6,29	-0,1	-0.15	-2,85	2,55	0.905
A N2 - Pós	-3,4	-9,46	-0,24				
L P3 - Pré	331,1	264	381	6.40	-18,19	30,99	0.571
L P3 - Pós	324,7	284	348				
A P3 - Pré	2,91	0,76	9,57	-1.82	-3,05	-0,58	**0.009**
A P3 - Pós	4,72	2,2	9,46				
DURAÇÃO P3 - Pré	104,4	55	142	-17.20	-28,34	-6,06	**0.007**
DURAÇÃO P3 - Pós	121,6	90	186				

**Legenda:** Dif = diferença; L = Latência; A = Amplitude; Pré = Pré intervenção; Pós = Pós intervenção; Li/Ls = limites do intervalo com 95% de confiança; Teste estatístico = Teste t para amostras emparelhadas

Os gráficos a seguir (Figura 3, Figura 4 e Figura 5) representam a comparação pré e pós da latência, amplitude e duração do PEALL no GC e no GE.

**Figura 3:** Comparação pré e pós dos valores de latência e amplitude dos potenciais P1 e N1 em ambos os grupos

**Legenda:** GC = Grupo controle; GE = Grupo estudo; L = Latência; A = Amplitude; Pré = Pré intervenção; Pós = Pós intervenção

**Figura 4:** Comparação pré e pós da latência e amplitude dos potenciais P2 e N2 em ambos os grupos

**Legenda:** GC = Grupo controle; GE = Grupo estudo; L = Latência; A = Amplitude; Pré = Pré intervenção; Pós = Pós intervenção

**Figura 5:** Comparação pré e pós dos valores de latência, amplitude e duração dos potencial P3 em ambos os grupos

**Legenda:** GC = Grupo controle; GE = Grupo estudo; L = Latência; A = Amplitude; Pré = Pré intervenção; Pós = Pós intervenção

Na Figura 6 é possível visualizar a representação gráfica da grande média dos PEALL pré e pós intervenção em ambos os grupos. A representação gráfica da grande média foi gerada a partir dos resultados da média dos componentes dos potenciais.

**Figura 6:** representação gráfica da grande média do PEALL pré e pós intervenção no GC e no GE

**Legenda:** GC = Grupo controle; GE = Grupo estudo; D = Duração

Já no GE, a duração e amplitude apresentaram mudanças estatisticamente significantes.

Ressalta-se que, nesta pesquisa, não houve ocorrência de eventos adversos.

## DISCUSSÃO

O presente estudo investigou os efeitos do TAM na percepção do zumbido e no PEALL, evidenciando redução significativa do incômodo e do impacto na qualidade de vida, além de alterações compatíveis com maior recrutamento e manutenção neural. Esses achados destacam o TAM como uma intervenção eficaz, capaz de promover adaptações funcionais nas áreas de audição e cognição por meio de estímulos que favorecem a neuroplasticidade auditiva^(20)^.

Os dados pré intervenção demonstram tudo que é necessário em pesquisas que comparam grupos em intervenções, destacando a homogeneidade dos dados para a comparação, trazendo total confiabilidade para as análises estatísticas. Além disso, pesquisadores ressaltam que os ensaios clínicos randomizados, duplo-cegos e com um grupo controle, são considerados o padrão-ouro para avaliar a eficácia de tratamentos e revelam mudanças clínicas fidedignas^(21)^.

Foram verificadas diferenças estatisticamente significantes nas comparações pré e pós intervenção em ambos os grupos para as variáveis EVA I, EVA V e questionário THI. Essas variáveis analisadas referem-se a padrões subjetivos de respostas dos indivíduos, e os resultados positivos em ambos os grupos podem estar associados ao acolhimento aos indivíduos e escuta ativa, o qual ocorreu tanto no grupo estudo quanto no no grupo que recebeu intervenção passiva. Porém ressalta-se que o GE apresentou uma mudança estatisticamente significante e expressiva em relação ao THI quando comparado com os resultados do GC, demonstrando a grande influência do TAM na qualidade de vida destes sujeitos.

Observou-se também que os participantes obtiveram benefícios com a intervenção, ampliando as opções de tratamento disponíveis para o zumbido ao incluir o TAM. Estudos anteriores envolvendo treinamento na modalidade auditiva-cognitiva também já demonstraram efeitos positivos no manejo deste sintoma^(20,22)^.

Esses resultados fortaleceram a ideia de que as intervenções ativas podem ter efeitos notáveis em várias condições clínicas, fato que justifica a melhora da percepção do sintoma nos indivíduos do presente estudo.

A melhora do sintoma no GC pode ser atribuída tanto à expectativa positiva gerada pelo simbolismo do tratamento quanto ao efeito da estimulação passiva, que envolveu exposição visual e auditiva, favorecendo a percepção do zumbido sem evocar emoções ou sentimentos negativos relacionados ao desconforto provocado pelo sintoma^(23)^. Embora os participantes tenham sido cegados durante a intervenção, ou seja, não tinham conhecimento sobre qual grupo estavam inseridos, os achados se alinham à literatura que aponta benefícios dessa modalidade pela crença na eficácia de uma suposta intervenção^(24)^. Ressalta-se que as atividades do grupo de estimulação passiva não apresentavam organização nem hierarquia das habilidades auditivas, o que indica que a melhora observada não decorreu de mecanismos de plasticidade do sistema auditivo, mas sim do acolhimento e do efeito positivo proporcionado pela experiência.

Ademais, quando comparadas as variáveis de percepção do sintoma por meio da escala EVA e do questionário THI nos momentos pré e pós entre os grupos, foi evidenciada maior redução da percepção de incômodo, volume e melhora do impacto na qualidade de vida no GE. Este fato possibilitou diferenças entre os grupos no momento pós intervenção. Este achado revela que o TAM foi mais eficaz em comparação à intervenção passiva. Sugere-se então, que a estimulação das habilidades auditivas por meio de estímulo musicais e associadas à estimulação cognitiva, estimula as regiões de córtex frontal e temporal. Ainda, impulsiona regiões de sistema límbico, englobando regiões auditivas e não auditivas afetadas em sujeitos que sofrem com o transtorno do zumbido^(25)^.

Esses resultados corroboram com o descrito por outros pesquisadores sobre o TAM, os quais referem que o mesmo traz benefícios na qualidade de vida, sintomas depressivos, aspectos cognitivos, resolução temporal e limitação em atividades de vida^(26,27)^. O TAM é baseado na musicalidade, o qual engloba mecanismos de processamento musical e sua conexão com processos bioquímicos, funcionais e fisiológicos do sistema auditivo, abrangendo aspectos auditivos, cognitivos e associações musicais^(28)^. Sendo assim, pode-se predizer que os resultados satisfatórios na percepção do sintoma e neuroplasticidade do GE estão associadas a estimulação auditiva e musical.

Quanto à mensuração dos efeitos neuroplásticos, na comparação dos resultados dos PEALL P1, N1, P2, N2 e P3, foi observada diferença estatisticamente significante para a variável duração do componente P3 no GC. No GE uma diferença estatisticamente significante para a variável duração e amplitude do componente P3, sendo que os valores médios aumentaram em ambos os grupos após o período de intervenção proposta para cada grupo.

A amplitude está relacionada à quantidade de recrutamento neural, enquanto a duração está relacionada à manutenção da atividade neural desempenhada^(29)^. O P3 é um potencial auditivo-cognitivo, ou seja, corresponde a habilidades auditivas desempenhadas e em relação a aspectos cognitivos como memória e atenção. Dessa forma, os resultados do presente estudo evidenciam melhora dos aspectos auditivos e atencionais no grupo que recebeu a intervenção por meio do TAM. Cabe destacar que a interpretação destes resultados levou em consideração a análise morfológica do componente P3 (Figura 2), considerando a análise conjunta da duração e amplitude do componente pré e pós intervenção.

Embora o GC tenha mostrado diferença na duração do componente entre os momentos pré e pós-intervenção, não houve aumento significativo no recrutamento neural. Isso indica que houve maior manutenção da atividade neural (refletida na maior duração do componente P3), sem aumento das fibras neurais recrutadas. Assim, pressupõe-se que a intervenção passiva teve efeitos limitados na organização neural dos sujeitos.

O GE aumentou o tempo da manutenção neural somado ao aumento considerável do recrutamento dos neurônios auditivos. Esse fato prediz sobre a melhora da atividade sináptica após o treinamento proposto. O aumento da amplitude somado à duração do P3 no GE também reflete a capacidade dos indivíduos perceberem com mais facilidade os estímulos verbais apresentados na realização do exame, evidenciando melhora dos aspectos de discriminação auditiva e habilidades atencionais. A estimulação auditiva desconecta o zumbido e direciona o foco do Sistema Nervoso Auditivo Central (SNAC), que alivia o sintoma ao promover a reorganização neural e realocação de cada atividade neural em sua função específica^(20,22)^.

A amplitude do P3 pode ser reduzida em indivíduos com zumbido, sugerindo que talvez haja uma inibição na atenção do estímulo externo em indivíduos com zumbido, sem modificações na latência^(30)^. No presente estudo não evidenciamos essa diferença para nenhuma variável eletrofisiológica na comparação entre os grupos pré intervenção, o que garantiu homogeneidade da amostra e reforça que os resultados eletrofisiológicos realmente são da intervenção.

Este estudo não revelou diferenças estatisticamente significativas na latência e amplitude dos componentes P1, N1, P2 e N2. Tal achado pode ser explicado pelo papel do zumbido como um estímulo adicional que afeta todos os componentes do PEALL. A emissão contínua de sinais aferentes gerados subcorticalmente pelo zumbido parece provocar uma adaptação no processamento dos sinais auditivos em geral. Essa adaptação sugere um mecanismo cerebral compensatório que pode influenciar os componentes do PEALL em pessoas com zumbido, já que esses potenciais estão relacionados tanto à atividade das vias auditivas quanto à atividade neuronal. Durante a aplicação do paradigma *Oddball*, é possível que indivíduos com zumbido percebam simultaneamente um estímulo frequente, o estímulo contínuo do zumbido e um estímulo raro e essa interação de estímulos pode dificultar a concentração no estímulo raro, gerando alterações na latência e na amplitude dos componentes dos potenciais de longa latência^(31)^.

De forma geral, a presente pesquisa evidencia que o TAM promove mudanças positivas na neuroplasticidade do sistema auditivo, e mudanças na percepção do sintoma. A aplicação do TAM confere ineditismo, pois não existem estudos na literatura que abordam os resultados de neuroplasticidade, visualizados através do PEALL, com a percepção do sintoma em sujeitos com transtorno do zumbido.

O trabalho longitudinal trouxe desafios, como encontrar sujeitos que se adequassem aos critérios de inclusão para intervenção em um curto período de tempo e também de oferecer uma intervenção passiva, seguido do TAM para todos sujeitos do GC. Apesar do estudo ter contemplado o número de sujeitos estabelecidos no cálculo amostral, cabe destacar que uma amostra maior seria adequada para verificar possíveis efeitos nos demais componentes dos PEALL, já que aumentaria o poder estatístico. Tal fato pode ser considerado uma limitação do estudo. Ainda, como possíveis limitações pode-se citar a possibilidade de viés de seleção, período de curta intervenção realizada, já que acredita-se que um número maior de sessões poderia impactar em melhores resultados e ausência de avaliações de seguimento a longo prazo.

Apesar disso, os resultados despertaram o interesse dos pesquisadores em dar continuidade ao estudo, considerando as dificuldades/limitações citadas. Para pesquisas futuras, recomenda-se o monitoramento audiológico dos indivíduos com transtorno de zumbido, visando avaliar os benefícios da intervenção a longo prazo.

Este estudo oferece evidências relevantes para a ciência e a prática clínica fonoaudiológica no manejo do zumbido, evidenciando que o TAM é uma técnica validada e estabelecida para ser aplicada, além de oferecer uma abordagem não invasiva e promissora para o manejo desse transtorno, e pode ser incorporado como estratégia complementar no manejo do zumbido em contexto clínico, embora mais estudos sejam necessários para confirmar sua efetividade a longo prazo.

## CONCLUSÃO

Por meio deste ensaio clínico, pode-se concluir que o treinamento auditivo musical (TAM) promoveu mudanças positivas na neuroplasticidade do sistema auditivo frente ao controle passivo. Além disso, observou-se uma redução na percepção e volume do transtorno do zumbido nos adultos participantes, refletindo em uma melhora significativa na qualidade de vida dos sujeitos.

Em relação às variáveis estudadas, o TAM promoveu melhora da duração e amplitude do componente auditivo-cognitivo P3, enquanto a intervenção passiva provocou mudanças positivas apenas na amplitude do P3, demonstrando o maior benefício da intervenção ativa.

Apesar das limitações do estudo, este artigo demonstra que o TAM é uma intervenção eficaz e disponível para ser implementada na prática clínica, oferecendo aos fonoaudiólogos uma estratégia inovadora e baseada em evidências para reduzir o zumbido e melhorar a função auditivo-cognitiva dos sujeitos que sofrem com este sintoma.
